# Supplementary material for: Understanding Trustful Relationships between Community Health Workers and Vulnerable Citizens during the COVID-19 Pandemic: A Realist Evaluation
Source: Int J Environ Res Public Health. 2022 Feb 22;19(5):2496. doi: 10.3390/ijerph19052496 (PMC8909775; doi:10.3390/ijerph19052496)
Supplement: Supplementary file 1 [file ijerph-19-02496-s001.zip › ijerph-1500216-supplementary.pdf]

Interview guide focus group discussions

1. What has it been like for you to guide and support a client during the lockdown measures? How did you experience this?
   - How did the contacts go? (What went well? What didn't go well? What did you find difficult?)
   - Tell us about an experience?
   - How did the first contact feel?
   - How did it feel when the guidance stopped?

2. What do you think we have achieved with these guidances? And what not?

3. What has this (the project and the guidance) meant to you?

4. What do you think this guidance has meant for your client?

5. Now suppose you were the client and you were accompanied by a volunteer. What would you have needed?

6. We have now done these guidances during the lockdown measures, but do you think it can also bring a change in the longer term?

7. Some general practitioners (GPs) gave the message that many guidances already exist. How did you experience this for your client?

8. Did you feel that you were sufficiently guided yourself?

9. After completing the online course, how did you feel then? Or what did you think then?
   a. How has that feeling evolved?
   b. What made a turning point in that feeling?

10. What makes you step into this project?

11. What have you told friends and family about this?
    a. What was their reaction?
    b. What did this do to you?

12. Would you do it differently afterwards?

13. Do you have any suggestions for those responsible?

Interview guide individual in-depth interviews

Interviews with CHWs

1. What has it been like for you to guide and support a client during the lockdown measures? How did you experience this?
    - How did the contacts go? (What went well? What didn't go well? What did you find difficult?)
    - Tell us about an experience?
    - How did the first contact feel?
    - How did it feel when the guidance stopped?

2. What do you think we have achieved with these guidances? And what not?

3. What has this (the project and the guidance) meant to you?

4. What do you think this guidance has meant for your client?

5. Now suppose you were the client and you were accompanied by a volunteer. What would you have needed?

6. We had expected that the social isolation and anxiety of the clients would decrease and that the feeling of emotional support and participating in activities would be improved. But we didn't see that based on our questionnaires. How did you experience that with your clients?

7. We have now done these guidances during the lockdown measures, but do you think it can also bring a change in the longer term?
    - If so, in what way/in what sense?
    - If not, can you tell us a bit more about that?

8. Some General practitioners (GPs) gave the message that so many guidances already exist. How did you experience this for your client? (Was there still a difference?)

9. Did you feel that you were sufficiently guided yourself? How did you experience the guidance by the coordinating team?

10. After completing the online course, how did you feel then? Or what did you think then?
    - How has that feeling evolved through the guidance?
    - What made a turning point in that feeling?

11. What makes you step into this project?

12. Was the task you were given as a volunteer in this corona project different for you from your work as a health guide/key figure/social guide?

13. What does it mean/does it mean that you have the same background as your client?

14. What have you told friends and family about this?
    - What was their reaction?
    - What did this do to you?

15. Would you do it differently afterwards?

16. Do you have any suggestions for those responsible?

Interviews with clients

1. How have the contacts with a volunteer been for you? How did you experience this?
    - How did the contacts go?
    - What did you do together?
    - For which matters could you call on the CHW?
    - What went well?
    - What didn't go so well?

2. How did it feel for you when the volunteer contacted you the first time? How has that evolved?

3. What did the contacts mean to you? What has this meant for you during corona?

4. What made you get into this project?

5. What did you think about your doctor knowing about this project?

6. What did other people think when you talked about this?

7. Would you participate again? What would you like to do differently?

8. Do you have any suggestions for those responsible?
